# Opioid prescribing practices in the U.S. and subsequent falls: a scoping review protocol

**DOI:** 10.1186/s13643-026-03150-9

**Published:** 2026-04-06

**Authors:** Viviane Isabelle Lovato, Maria Rocío Torres, Deborah Jean McClelland, Jeannie K. Lee, Abdul Tawab Saljuqi, Bridget S. Murphy

**Affiliations:** 1 Arizona Center for Rural Health, Mel & Enid Zuckerman College of Public Health, The University of Arizona, 1295 N Martin Ave, Tucson, AZ 85719 USA; 2 Health Sciences Library, The University of Arizona, Tucson, AZ USA; 3 Department of Pharmacy Practice & Science, R. Ken Coit College of Pharmacy, Division of General Internal Medicine, Geriatrics & Palliative Medicine, College of Medicine-Tucson, The University of Arizona, Tucson, AZ USA; 4 The Division of Trauma, Surgical Critical Care, Burns, and Acute Care Surgery, College of Medicine, Mel & Enid Zuckerman College of Public Health, The University of Arizona, Tucson, AZ USA; 5 Department of Health Promotion Sciences, Mel & Enid Zuckerman College of Public Health, Comprehensive Center for Pain and Addiction, The University of Arizona, Tucson, AZ USA

**Keywords:** Chronic pain, Opioids, Prescribing, Falls

## Abstract

**Background:**

Falls resulting from or related to opioid use are a major concern for morbidity and mortality in the United States. The Centers for Disease Control and Prevention’s Clinical Practice Guideline for Prescribing Opioids for Pain – United States 2022 seeks to enhance the effectiveness and safety of pain treatment by improving function and quality of life for patients with pain while reducing opioid-related risks. The American College of Surgeons’ Best Practices Guidelines in Geriatric Trauma Management and the 4 M’s Framework of an Age-Friendly Health System both recommend incorporating fall prevention and safe mobility strategies into healthcare for older adults. In line with these guidelines and our review questions, this scoping review protocol aims to better understand the research on opioid prescribing practices and subsequent falls by identifying and mapping (1) the types of available evidence about opioid prescribing practices and subsequent falls and (2) the key characteristics of treatment and continuing care of falls with specific emphasis on opioid prescribing practices.

**Methods:**

Electronic database searches will be conducted in PubMed, Embase, CINAHL, and Scopus to identify evidence published in the United States from January 1, 2016, onward. This scoping review will consider primary literature, observational, quasi-experimental, and experimental, implementation, and quality improvement studies, as well as systematic reviews and meta-analyses. Eligible studies will focus on adults aged 18 years and older who have been prescribed opioids and subsequently experienced a fall.

**Discussion:**

There is a gap in our understanding about opioid prescribing and subsequent falls. This scoping review will help fill this gap by mapping the recent available evidence—aligned with clinical guidelines about (1) prescribing practices and subsequent falls and (2) key characteristics of the treatment and continuing care of falls.

**Systematic review registration:**

Open Science Framework ID: 10.17605/OSF.IO/JP49Y. https://osf.io/jp49y.

**Supplementary Information:**

The online version contains supplementary material available at 10.1186/s13643-026-03150-9.

## Background

In the United States (U.S.), chronic pain is experienced by 20% of the population and it is a primary reason patients seek care. Global evidence shows that opioids increase the risk and occurrence of accidental injury, especially falls [1–7]. Adverse effects of opioids increase the risk of falls through drowsiness, dizziness, fatigue, impaired cognition, low blood pressure, decreased sodium levels in the blood, and effects on the central nervous system [2, 5, 8]. Falls are the most common cause of traumatic brain injury (TBI) and are a significant concern for older adults [9]. One in four individuals aged 65 years and older experiences a fall each year, and one in ten falls results in an injury that restricts mobility [9]. This costs over $50 billion each year in medical expenses in the United States (U.S.) [10]. While the majority of this evidence comes from studies on falls in older adults conducted outside the U.S., an exploration of scientific literature in the U.S. on opioids and workplace injuries found that “both opioid and benzodiazepine medications have been associated with increased risks of falls” ([5] p. 199).

### Pain and opioids

A systematic review of 14 studies encompassing more than 17,000 older adults reported that pain was associated with a statistically significant 56% increase in the odds of falling [11]. The International Association for the Study of Pain (IASP) defines pain as “an unpleasant sensory and emotional experience associated with actual or potential tissue damage or described in terms of such damage” ([11] p. 2). Chronic pain prevention, assessment, and management remain persistent challenges for clinicians due to its multifactorial nature. Pain lacks objective biological markers and is influenced by interacting with biological, psychological, and social factors, as well as variations in clinical practice and gaps in care [7].

Consistent with this complexity, a study examining unmet mental health needs in adults with chronic pain used a National Health Interview Survey (NHIS) question on the amount of pain experienced over time to determine if chronic pain was present, aligning with definitions from both the IASP and the International Classification of Diseases (ICD-10) [12]. While opioids are used in the treatment of pain, in 2020, prescription opioids remained the most commonly misused prescription medication in the U.S. [7]. Among individuals reporting prescription opioid misuse that year, 64.6% cited relief of physical pain as the primary reason for their most recent misuse, compared with 11.3% who reported use to “feel good or get high” and 2.3% who reported misuse due to dependence [7].

### Centers for Disease Control and Prevention’s Clinical Practice Guideline for Prescribing Opioids for Pain

In 2016, the Centers for Disease Control and Prevention (CDC) created the first version of the CDC Clinical Practice Guideline for Prescribing Opioids for Pain (hereafter referred to as the CDC Guideline). The CDC Guideline is a clinical tool that provides evidence-based recommendations designed to be flexible in supporting person-centered, collaborative decision-making between clinicians and patients [7].

Clinical decisions are guided by individual clinical indications and patients’ anticipated health outcomes and overall well-being. Where appropriate, family considerations may also be taken into account [7]. The overarching goal is to improve the effectiveness and safety of pain treatment by enhancing the ability to function and quality of life for patients with pain and reducing risks associated with opioids [7].

This guideline is intended for use by clinicians providing outpatient pain care to adults aged 18 years and older with acute (<1 month), subacute (1–3 months), and chronic (>3 months) pain [7]. In 2022, the CDC published an updated version of the guideline, which expanded the scope and refined the focus of 12 recommendations grouped into four areas: (1) determining whether or not to initiate opioids for pain, (2) selecting opioids and determining dosages, (3) deciding duration of initial opioid prescription and conducting follow-up, and (4) assessing risk and addressing potential harms of opioid use [7].

### Best practice guidelines for falls

When considering clinical practices around falls, it is important to note that falls are not classified as injuries themselves but rather as causes of injury. Within the International Classification of Diseases, Tenth Revision (ICD-10), falls are categorized under *External Causes of Morbidity*, specifically codes W00–W19, which are not mandated for national reporting [12, 13]. As a result, the consistent documentation and surveillance of falls are challenging, and the ability to accurately assess their occurrence and associated outcomes is limited. ICD-10 codes W00–W19 encompass a broad range of fall mechanisms, including slipping, tripping, and stumbling, as well as falls involving ice or snow, furniture, stairs, playground equipment, and other specified or unspecified causes (see Appendix 1 for a complete description) [12]. For ground-level falls (W01), particularly among older adults, multiple high-quality clinical guidelines and recommendations are available in the U.S. to inform fall prevention practices across healthcare settings [12, 14–18].

This scoping review will focus on the American College of Surgeons (ACS) Best Practices Guidelines in Geriatric Trauma Management (hereafter referred to as the ACS Guidelines) and the Institute for Healthcare Improvement’s (IHI) 4Ms Framework of an Age-Friendly Health System (hereafter referred to as the 4Ms Framework) [18, 19]. Both the ACS Guidelines and the 4Ms Framework provide recommendations for medication management and prescribing practices for older adults [18–20]. The ACS Guidelines contain four key areas: (1) emphasis on the need for prevention of falls, (2) tailored geriatric criteria, (3) interdisciplinary approach, and (4) emphasis on the need for gentle care [16]. Similarly, the 4Ms Framework encompasses four significant elements for providing optimal care to older adults in various health systems: (1) *What Matters*—aligning care with what matters to the patient, (2) *Medication*—using age-appropriate medications that do not interfere with the other three M’s, (3) *Mentation*—addressing mental health and cognition, specifically dementia, depression, and delirium, and (4) *Mobility*—ensuring that older adults can safely do what matters to them [18, 19].

### Opioids and falls

Adverse effects associated with prescription drugs are one of the most important modifiable risk factors for falls. Given the evidence of opioids and increased fall risk, opioid prescribers should consider the potential for subsequent falls when making prescribing decisions [6]. However, a Canadian review published in 2012 found no consistent evidence supporting the existence of effective interventions to address opioid use and subsequent falls [6]. To better understand the recent research on opioid prescribing practices and subsequent falls, we propose this scoping review to identify and map (1) types of available evidence about opioid prescribing practices and subsequent falls and (2) key characteristics of the treatment and continuing care of falls with specific emphasis on opioid prescribing practices. Furthermore, we will focus on U.S.-based evidence to better understand how these prescribing practices align with the U.S.-based guidelines (see Table 1). To our knowledge, no similar reviews with our specific focus on opioid prescribing practices and the occurrence of subsequent falls within the U.S. context have been or are currently being conducted. While prior reviews have explored the relationship between opioid use and fall risk, these have primarily focused on older adult populations or the effects of multiple medication classes, rather than on opioid prescribing practices themselves. As such, this scoping review addresses an important gap in literature.

**Table 1:** Guidelines for opioid prescribing and falls prevention

**Guideline**	Centers for Disease Control and Prevention’s (CDC) Clinical Practice Guideline for Prescribing Opioids for Pain	American College of Surgeons (ACS) Best Practices Guidelines in Geriatric Trauma Management	Institute for Healthcare Improvement 4Ms Framework of an Age Friendly Health System
**Focus areas**	Area 1 – Determining whether or not to initiate opioids for pain	Area 1 – Emphasis on the need for falls prevention	Area 1—What Matters—Aligning care with what matters to the patient
	Area 2 – Selecting opioid agents and determining dosages	Area 2 – Tailored geriatric criteria	Area 2—Medication—Using age-friendly medication that does not interfere with the other three M’s
	Area 3 – Deciding the duration of the initial opioid prescription and conducting follow-up	Area 3—Interdisciplinary approach	Area 3—Mentation—Addressing mental health, specifically dementia, depression, and delirium
	Area 4 – Assessing risk and addressing potential harms for opioid use	Area 4 – Emphasis on the need for gentle care	Area 4—Mobility—Ensure that older adults can safely do what matters to them
**Setting**	Clinical	Clinical	Community, public health and clinical
**Population**	18 years of age or older	Geriatric	Older adults

### Guiding theoretical model

**Fig. 1:**
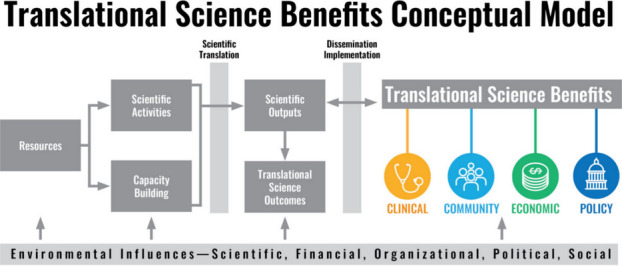
Translational Science Benefits Model. Creative Commons Attribution-Noncommercial-Share Alike 4.0 International License

The aim of Clinical and Translational Science (CTS) is to accelerate the translation of research innovations into practice by using evidence-based interventions to inform implementation strategies. The Translational Science Benefits Model (TSBM) assesses health and societal impacts in four domains, including clinical, community, economic, and policy (see Figure 1 in Appendix 2) [21, 22]. Researchers and scholars argue that using the TSBM to assess the benefits of research enhances the uptake of innovations [23]. TSBM will serve as our guiding theoretical model, and we will categorize results in the four domains to identify benefits of practice guidelines, areas for improvement, gaps in literature, and opportunities for CTS studies.

## Review questions

Broadly, we want to identify and map the available evidence related to opioid prescribing and subsequent falls.

#1 Identify and map the types of available evidence about opioid prescribing practices and subsequent falls.

- ✦ What types of research exist that address opioid prescribing and subsequent falls (study type, setting, population)?
- ✦ What types of falls, classified under ICD-10 codes W00–W19 (including falls due to slipping, tripping, stumbling, and falls from or due to ice, snow, furniture, stairs, playground equipment, or unspecified causes; see Appendix 1 for a complete description), have been examined for their association with opioid use? 
- ✦ What have been the primary outcomes/endpoints of existing studies?
- ✦ Which opioid medications are most often prescribed that are associated with falls?
- ✦ Who are the prescribers (e.g., profession type and specialty)?
- ✦ What types of clinical decision-making technologies are used to support pain management, including opioid prescribing practices?

#2 Identify and map key characteristics of the treatment and continuing care of falls with specific emphasis on opioid prescribing practices.

- ✦ How are falls and opioid prescribing practices aligned with the CDC Clinical Practice Guideline for Prescribing Opioids for Pain? (TSBM clinical)
  -
    - ◦ Area 1 – Determining whether or not to initiate opioids for pain
    - ◦ Area 2 – Selecting opioid agents and determining dosages
    - ◦ Area 3 – Deciding the duration of the initial opioid prescription and conducting follow-up
    - ◦ Area 4 – Assessing risk and addressing potential harms for opioid use
- ✦ How are falls and opioid prescribing practices aligned with the ACS Best Practices Guidelines in
  Geriatric Trauma Management? (TSBM clinical)
  -
    - ◦ Area 1 – Emphasis on the need for falls prevention
    - ◦ Area 2 – Tailored geriatric criteria
    - ◦ Area 3 – Interdisciplinary approach
    - ◦ Area 4 – Emphasis on the need for gentle care
- ✦ How are falls and opioid prescribing practices aligned with the 4 M’s Framework of an Age-Friendly Health System (TSBM community, public health and clinical)
  -
    - ◦ Area 1 – What Matters – Aligning care with what matters to the patient
    - ◦ Area 2 – Medication – Using age-friendly medication that does not interfere with the other three M’s
    - ◦ Area 3 – Mentation – Addressing mental health, specifically dementia, depression, and delirium
    - ◦ Area 4 – Mobility – Ensure that older adults can safely do what matters to them
- ✦ What are the differential characteristics of the populations that have access to care: i.e., age, sex/gender/gender identity, education, race/ethnicity, language, traditions, beliefs, education levels, income levels, insurance, state, geography (acute vs. chronic pain; types of pain/prescribing indication) (duration of medication use) (TSMB community/public health)?
- ✦ Who is on the treatment team (e.g., interprofessional teams) (TSBM clinical)?
- ✦ Are falls prevention referrals provided? (what programs and who made the referral) (TSBM clinical, community/public health)
- ✦ What is known about the costs/benefits associated with falls and opioid prescribing? (TSBM economics)
- ✦ What is known about the prescribing policies that affect treatment of falls and opioid prescribing? (TSBM policies)
- ✦ Does the manuscript provide practitioner, public health, policy, or financial recommendations?

## Methods

The proposed scoping review will be conducted in accordance with the Joanna Briggs Institute (JBI) methodology for scoping reviews [24] and the Preferred Reporting Items for Systematic Reviews and Meta-Analyses extension for Protocols (PRISMA-P) 2015 checklist [25] (see the PRISMA-P checklist in Additional file 1). This scoping review has been registered with the Open Science Framework ID: 10.17605/OSF.IO/JP49Y.

### Inclusion criteria

#### Population

This scoping review will focus on adults aged 18 years or older who are prescribed opioids and experience a subsequent fall. All patient sociodemographic characteristics will be included. To ensure alignment with U.S.-based guidelines (i.e., the CDC and ACS Guidelines, and the 4Ms Framework), only studies conducted in the U.S. will be included.

#### Concept

This scoping review will include research on opioid prescribing practices and the occurrence of subsequent falls, encompassing research with correlational, causal, or observational designs. Studies conducted across all levels of care, from primary to specialty care, will be included. To be eligible for inclusion, studies must specifically address both opioid prescribing and falls.

#### Context

This review will focus on studies conducted in the U.S. and published on or after January 1, 2016. The start date aligns with the publication of the first CDC Guideline for Prescribing Opioids for Pain, and the review will reference the updated 2022 CDC Guideline. Additionally, the 4Ms Framework has emerged within the past 10 years, and the most recent ACS Guidelines were published in 2023. No language restrictions will be applied; studies published in languages other than English will be translated as needed to ensure inclusion (as long as they are based in the U.S.).

### Search strategy

The search strategy was developed in collaboration with the medical librarian on the review team. Electronic searches will be conducted in the following databases: PubMed (National Center for Biotechnology Information), Embase (Elsevier), Cumulative Index to Nursing and Allied Health Literature (CINAHL (EBSCO)), and Scopus (Elsevier). The search strategy developed for PubMed incorporates three core concepts: (1) prescription opioids, (2) prescribing practices, and (3) subsequent falls and is presented in Appendix 3. This PubMed search includes both keywords and corresponding Medical Subject Headings (MeSH) terms and will be translated to the other three databases, including controlled vocabulary (e.g., Emtree).

EndNote 25 (Clarivate) will serve as the initial repository for all references found in the electronic searches [26]. Resulting records in each database will be deduplicated using EndNote 25, after which the final deduplicated set of records will be imported into the Rayyan web-based research collaboration platform for screening against prespecified inclusion criteria [26, 27]. EndNote will also be utilized as a citation tool for the scoping review report [26].

#### Types of sources

This scoping review will consider a broad range of evidence sources, including primary, observational, quasi-experimental, experimental, implementation, and quality improvement (QI) studies, as well as systematic reviews and meta-analyses.

#### Grey literature exclusion justification

While we realize there is a potential for bias in excluding grey literature, we have decided to exclude it for two related reasons: (1) the opioid epidemic has generated an enormous amount of literature that could be categorized as grey (e.g., blogs; catalogs, annual reports, presentations, books, government reports), and (2) the capacity of our research team. We are using falls and opioid prescribing practice-based guidelines to focus our review, and we will document the articles that provide relevant findings for non-academics.

### Source of evidence selection

Selected records will be uploaded to the Rayyan web-based research collaboration platform to facilitate and streamline the systematic review process [27, 28]. Sources will be screened in two stages: initial title and abstract screening, followed by full-text review of potentially relevant articles. Title and abstract screening will be conducted by junior subject matter reviewers under the guidance of at least one senior subject matter investigator. The reference lists of included articles will be searched for critical review by junior reviewers to identify additional eligible sources.

Full-text screening will be performed by junior-level reviewers, and two investigators will review against the predefined inclusion criteria prior to the data extraction step. Any excluded articles will be recorded and reported in the review with explanations. Final results of the search process, including identification, screening, full-text review, and inclusion of final studies, will be documented through the PRISMA-P flow chart.

Any discrepancies arising during the screening process will be resolved between the two junior reviewers to achieve consensus. If there are remaining differences, a senior investigator will serve as an arbiter. Junior-level staff/collaborator involvement in the review process will provide an opportunity to develop foundational knowledge and practical skills in conducting scoping reviews, including study screening, data extraction, and the synthesis and presentation of results. Junior reviewers to be all encompassing of the diversity of levels of people included.

#### Pilot testing

Prior to commencing source selection, pilot testing will be undertaken for both the screening and data extraction processes to refine the review methodology and associated tools. An initial pilot screening of identified sources will involve a sample of 100 titles and abstracts, which will be independently reviewed against predefined inclusion criteria by two junior reviewers under the guidance of at least one senior investigator. Any discrepancies in screening results will be discussed among the two junior reviewers and at least one investigator to reach at least 75% agreement. Further discussions about inclusion criteria and review questions will take place if the threshold is not met. Reviewers will continue screening identified sources until the established agreement level is reached, and duplicate records will be removed.

To ensure the effectiveness of the data extraction tool (Appendix 4), pilot testing will be conducted using two sources selected for full-text review. For this pilot test, one junior reviewer will independently extract data using the extraction instrument template, and a second junior reviewer will verify the consistency, accuracy, and completeness of the extracted data. The results of the pilot test will be discussed among all reviewers, and any necessary refinements to the data extraction tool will be made accordingly. All modifications, along with the final version of the data extraction tool, will be documented and reported in the completed scoping review.

### Data extraction

For data extraction, two junior reviewers will independently use a modified JBI template source of evidence details, characteristics, and results extraction instrument to collect data from all included sources (Appendix 4). All modifications to the JBI instrument were discussed among the research team to ensure alignment with the context and research questions of this scoping review. Any disagreements regarding study inclusion or exclusion arising during the data extraction process will be resolved through discussion between the two junior reviewers to achieve consensus; unresolved disagreements will be adjudicated by a third senior reviewer. The data extraction process may be iteratively refined as familiarity with the evidence base increases; however, no changes will be made to the predefined inclusion or exclusion criteria. All modifications to the data extraction process will be fully documented and reported in the final scoping review.

Consistent with JBI guidance for scoping reviews, formal critical appraisal and risk of bias assessment of individual sources of evidence will not be conducted, as the purpose of this review is to map the breadth of existing literature rather than to evaluate the effectiveness or certainty of the evidence required in a systematic review and meta-analysis.

### Data analysis and presentation

Data will be presented using descriptive tables and graphical formats, including bar graphs, pie charts, mapping, heatmaps, and/or line graphs, as appropriate. Each graphic will include a description and will be explicitly referenced within the scoping review text. Each review question and its corresponding findings will be presented in the report. Conceptual categories for data presentation will include evidence type; study population characteristics (age, race/ethnicity, and environment [rural/urban]; study setting; services/interventions provided; types of falls classified using ICD-10 codes W00-W19; alignment with relevant guidelines and their focus areas; and identified opportunities for CTS studies.

Additionally, medications prescribed for pain management and for opioid use disorders will be identified and categorized separately, recognizing that certain medications (e.g., methadone and buprenorphine) may be indicated for both purposes. These categories may be adjusted to enhance effectiveness and clarity before finalizing the scoping review; any modifications and their rationale will be transparently documented. Key findings and gaps in the existing literature will be summarized and discussed with relevant tables and figures.

## Discussion

In the context of the ongoing opioid epidemic, the appropriate use of opioids as a legitimate modality for pain management remains critically important. The development of experience- and evidence-based clinical guidelines by the CDC represents a substantial advancement in addressing opioid prescribing practices. Nevertheless, despite, or perhaps because of, these improvements, significant questions persist regarding the optimal use of opioids and the mitigation of their unintended consequences. This scoping review seeks to examine these issues by documenting best practice strategies for addressing pain, opioid prescribing, and mitigating subsequent risks, specifically the occurrence of falls.

Although the review questions are informed by three U.S.-based clinical guidelines and the TSBM theoretical model [7, 16–23], several limitations must be acknowledged. Consistent with JBI guidance, this scoping review will not include a formal critical appraisal of methodological quality or a structured assessment of risk of bias using validated tools. As such, the review is intended to map the breadth and nature of the existing evidence rather than to evaluate health outcomes or establish causal relationships.

Despite these limitations, this scoping review addresses an important gap in the literature by systematically examining opioid prescribing practices in relation to subsequent falls within the U.S. and may help inform systematic reviews, future research, public health, and clinical practice.

## Supplementary Information

Additional file 1. PRISMA-P 2015 Checklist.

## Data Availability

Our search will be conducted in publicly accessible electronic databases: PubMed (National Center for Biotechnology Information), Embase (Elsevier), Cumulative Index to Nursing and Allied Health Literature (CINAHL (EBSCO)), and Scopus (Elsevier).

## Appendix 1

### The International Classification of Diseases (ICD-10) Codes for Falls under External Causes for Morbidity W00–W19

**Table:**

W00	Fall due to ice and snow
W01	Fall on same level from slipping, tripping and stumbling
W03	Other fall on same level due to collision with another person
W04	Fall while being carried or supported by other persons
W05	Fall from non-moving wheelchair, nonmotorized scooter and motorized mobility scooter
W06	Fall from bed
W07	Fall from chair
W08	Fall from other furniture
W09	Fall on and from playground equipment
W10	Fall on and from stairs and steps
W11	Fall on and from ladder
W12	Fall on and from scaffolding
W13	Fall from, out of or through building or structure
W14	Fall from tree
W15	Fall from cliff
W16	Fall, jump or diving into water
W17	Other fall from one level to another
W18	Other slipping, tripping and stumbling and falls
W19	Unspecified fall

2025 ICD-10-CM Codes W00–W19: Slipping, tripping, stumbling, and falls from: https://www.icd10data.com/ICD10CM/Codes/V00-Y99/W00-W19

## Appendix 3

### Search strategy

#### Prescribed Opioids and Subsequent Falls: Scoping Review Protocol- PubMed

(“Wounds and Injuries/etiology”[MeSH] OR “Analgesics, Opioid/adverse effects”[MeSH] OR “Accidental Falls”[MeSH] OR falls [tiab] OR falling [tiab] OR fall [tiab])

AND

(“Pain, Postoperative/drug therapy”[MeSH] OR “Drug Prescriptions/statistics and numerical data”[MeSH] OR “Patient Discharge”[MeSH] OR “Analgesics, Opioid/therapeutic use”[MeSH] OR “Analgesics, Opioid/administration and dosage”[MeSH] OR opioid prescription [tiab] OR prescription [tiab] OR prescriptions [tiab] OR prescribed [tiab] OR prescribe [tiab]OR prescriber [tiab] OR prescribing [tiab])

AND

(“Analgesics, Opioid”[MeSH] OR “Narcotics”[MeSH] OR “Buprenorphine”[MeSH] OR Buprenorphine[tiab] OR Belbuca[tiab] OR Butrans[tiab] OR Sublocade[tiab] Suboxone[tiab] OR Zubsolv[tiab] OR Buprenex[tiab] OR Sublocade[tiab] OR “Buprenorphine, Naloxone Drug Combination”[Mesh] OR Bunavail[tiab] OR “Butorphanol”[MeSH] OR Butorphanol[tiab] OR Stadol[tiab] OR “Codeine”[MeSH] OR Codeine[tiab] OR “Fentanyl”[MeSH] OR Fentanyl[tiab] OR Actiq[tiab] OR Duragesic[tiab] OR Fentora[tiab] OR Lazanda[tiab] OR Sublimaze[tiab] OR Abstral[tiab] OR Ionsys[tiab] OR Subsys[tiab] OR “Hydrocodone”[Mesh] OR “Hysingla ER”[tiab] OR “Zohydro ER”[tiab] OR Lorcet[tiab] OR Lortab[tiab] OR Norco[tiab] OR Vicodin[tiab] OR Vicoprofen[tiab] OR “Hydromorphone”[MeSH] OR hydromorphone[tiab] OR Dilaudid[tiab] OR Exalgo[tiab] OR “Levorphanol”[Mesh] OR Levorphanol[tiab] OR “Levo-Dromoran”[tiab] OR “Meperidine”[MeSH] OR Meperidine[tiab] OR Demerol[tiab] OR “Methadone”[MeSH] OR Methadone[tiab] OR Dolophine[tiab] OR Methadose[tiab] OR “Morphine”[Mesh] OR Morphine[tiab] OR Duramorph[tiab] OR Infumorph[tiab] OR Mitigo[tiab] OR “MS Contin”[tiab] OR “Arymo ER”[tiab] OR Kadian[tiab] OR MorphaBond[tiab] OR “morphine, naltrexone combination”[Supplementary Concept] OR “Embeda”[tiab] OR “Naltrexone”[Mesh] OR naltrexone[tiab] OR “Oxycodone”[MeSH] OR Oxycodone[tiab] OR Oxycontin[tiab] OR Oxaydo[tiab] OR Roxicodone[tiab] OR RoxyBond[tiab] OR “Xtampza ER”[tiab] OR Endocet[tiab] OR Percocet[tiab] OR Roxicet[tiab] OR “Aspirin-oxycodone hydrochloride-oxycodone terephthalate combination”[Supplementary Concept] OR Percodan[tiab] OR “Oxymorphone”[Mesh] OR Oxymorphone[tiab] OR Opana[tiab] OR “Tapentadol”[Mesh] OR tapentadol[tiab] OR Nucynta[tiab] OR “Tramadol”[MeSH] OR Tramadol[tiab] OR Conzip[tiab] OR Ultram[tiab] OR Ultracet[tiab])

## Appendix 4

### Modified JBI template source of evidence details, characteristics, and results extraction instrument

**Table:**

**Scoping Review Details**	
Scoping Review title:	Opioid Prescribing Practices in the U.S. and Subsequent Falls: A Scoping Review Protocol
Review objective/s:	Identify and map the available evidence #1) The types of available evidence about opioid prescribing practices and subsequent falls #2) The key characteristics of the treatment and continuing care of falls with specific emphasis on opioid prescribing practices.
Review question/s:	Objective #1 Review Questions ▪ What types of research exist that address opioid prescribing and subsequent falls (study type, setting, population)? ▪ What types of falls, classified under ICD-10 codes W00–W19 (including falls due to slipping, tripping, stumbling, and falls from or due to ice, snow, furniture, stairs, playground equipment, or unspecified causes), have been examined for their association with opioid use? ▪ What have been the primary outcomes/endpoints? ▪ Who are the prescribers (e.g., profession type and specialty)? ▪ What types of clinical decision-making technologies are used to support pain management, including opioid prescribing practices? Objective #2 Review Questions ▪ How are falls and opioid prescribing practices aligned with the* CDC Clinical Practice * *Guideline for Prescribing Opioids for * *Pain?* (TSBM clinical) o Area 1 – Determining whether or not to initiate opioids for pain o Area 2 – Selecting opioid agents and determining dosages o Area 3 – Deciding the duration of the initial opioid prescription and conducting follow-up o Area 4 – Assessing risk and addressing potential harms for opioid use ▪ How are falls and opioid prescribing practices aligned with the ACS *Best Practices * *Guidelines in Geriatric Trauma * *Management*? (TSBM clinical) o Area 1 – Emphasis on the need for falls prevention o Area 2 – Tailored geriatric criteria o Area 3 - Interdisciplinary approach o Area 4 – Emphasis on the need for gentle care ▪ How are falls and opioid prescribing practices aligned with the 4M’s Framework of an Age-Friendly Health System(TSBM community, public health & clinical) o Area 1 - What Matters - Aligning care with what matters to the patient o Area 2 - Medication - Using age friendly medication that doesn’t interfere with the other three M’s o Area 3 - Mentation - Addressing mental health, specifically dementia, depression, and delirium o Area 4 - Mobility - Ensure that older adults can safely do what matters to them ▪ How are culturally and linguistically responsive services integrated? ▪ What are the differential characteristics of the populations that have access to care: i.e. age, sex/gender/gender identity, education, race/ethnicity, education levels, income levels, insurance, state, geography (acute vs. chronic pain; types of pain/prescribing indication) (duration of medication use) (TSMB community/public health)? ▪ Who is on the treatment team (e.g., interprofessional teams) (TSBM clinical)? ▪ Are falls prevention referrals provided? ▪ What is known about the costs/benefits associated with falls and opioid prescribing? (TSBM economic) ▪ What is known about the prescribing policies that affect treatment of falls and opioid prescribing? (TSBM policies) ▪ Does the manuscript provide practitioner, public health, policy, or financial recommendations?
**Inclusion/Exclusion Criteria**	
Population	U.S.; adult population; 18 years of age or older
Concept	Opioids prescribing habits and the occurrence of subsequent falls
Context	No language restrictions Date limit published on or before Jan 1, 2016, and forward
Types of evidence source	Primary, observational, quasi-experimental, experimental, implementation, and quality improvement (QI) studies; systematic reviews and meta-analyses reviews
**Evidence source Details and Characteristics**	
Citation details	
Country	
Context/Setting	
Participants/population (with access to care)	
**Details/Results extracted from source of evidence**	
Type of evidence source	
Types of falls (W00-W19)	
Primary outcomes/endpoints	
Type of clinical decision-making technology used to support pain and opioid prescribing practices	
Alignment with 2022 CDC Guideline	
Alignment with ACS Guidelines	
Alignment with 4Ms Framework	
Details of culturally and linguistically responsive services integrated	
Details of falls prevention referrals	
Costs/benefits associated with falls and opioid prescribing	
Details of prescribing policies that affect treatment of falls and opioid prescribing	
Practitioner, public health, policy, or financial recommendations	

Modified Appendix 10.1 from: Peters MDJ, Godfrey C, McInerney P, Munn Z, Tricco AC, Khalil H. Scoping Reviews (2020). Aromataris E, Lockwood C, Porritt K, Pilla B, Jordan Z, editors. JBI Manual for Evidence Synthesis. JBI; 2024. Available from: https://synthesismanual.jbi.global.

https://jbi-global-wiki.refined.site/space/MANUAL/355862497/10.+Scoping+reviews.

## Abbreviations

ACS: American College of Surgeons
CDC: Centers for Disease Control and Prevention
CINAHL: Cumulative Index to Nursing and Allied Health Literature
CTS: Clinical and Translational Science
IASP: International Association for the Study of Pain
ICD-10: International Classification of Diseases
IHI: Institute for Healthcare Improvement
JBI: Joanna Briggs Institute
MeSH: Medical Subject Headings
NHIS: National Health Interview Survey
PRISMA-P: Preferred Reporting Items for Systematic Review and Meta-Analysis Protocols
QI: Quality improvement
TBI: Traumatic brain injury
The 4Ms Framework: What Matters Most, Medication, Mentation, and Mobility
TSBM: Translational Science Benefits Model
U.S.: The United States
